# The expression of the ubiquitin ligase SIAH2 (seven *in absentia *homolog 2) is mediated through gene copy number in breast cancer and is associated with a basal-like phenotype and p53 expression

**DOI:** 10.1186/bcr2828

**Published:** 2011-02-09

**Authors:** Peter Chan, Andreas Möller, Mira CP Liu, Jaclyn E Sceneay, Christina SF Wong, Nic Waddell, Katie T Huang, Alexander Dobrovic, Ewan KA Millar, Sandra A O'Toole, Catriona M McNeil, Robert L Sutherland, David D Bowtell, Stephen B Fox

**Affiliations:** 1 Department of Pathology, Peter MacCallum Cancer Centre, Locked Bag 1 A'Beckett Street, Melbourne, Victoria 8006, Australia; 2 Research Division, Cancer Genetics and Genomics, Peter MacCallum Cancer Centre, Locked Bag 1 A'Beckett Street, Melbourne, Victoria 8006, Australia; 3 Department of Pathology, University of Melbourne, Royal Parade, Parkville, Victoria 3010, Australia; 4 Queensland Centre for Medical Genomics, Institute for Molecular Bioscience, University of Queensland, Brisbane, Queensland 4072, Australia; 5 Cancer Research Program, Garvan Institute of Medical Research, 384 Victoria Street, Darlinghurst, NSW 2010, Australia; 6 South East Area Laboratory Services, St George Hospital, Kogarah, NSW 2217, Australia; 7 School of Medicine, University of Western Sydney, Campbelltown NSW 2751, Australia; 8 School of Medical Sciences, Faculty of Medicine, University of New South Wales, Sydney, NSW 2052, Australia; 9 Department of Tissue Pathology and Diagnostic Oncology, Royal Prince Alfred Hospital, Camperdown, NSW 2006, Australia; 10 Faculty of Medicine, University of Sydney, NSW 2006, Australia; 11 Department of Medical Oncology & Breast Cancer Institute of New South Wales, University of Sydney, Westmead Hospital, Westmead, NSW 2145, Australia; 12 Western Clinical School, University of Sydney, Westmead Hospital, Westmead, NSW 2145, Australia

## Abstract

**Introduction:**

The seven *in absentia *homolog 2 (SIAH2) protein plays a significant role in the hypoxic response by regulating the abundance of hypoxia-inducible factor-α; however, its role in breast carcinoma is unclear. We investigated the frequency and expression pattern of SIAH2 in two independent cohorts of sporadic breast cancers.

**Methods:**

Immunohistochemical evaluation of SIAH2protein expression was conducted in normal breast tissues and in tissue microarrays comprising ductal carcinoma *in situ *(DCIS) and a cohort of invasive breast carcinomas. Correlation analysis was performed between SIAH2 and clinicopathological variables and intrinsic breast cancer subgroups and validated in a cohort of 293 invasive ductal carcinomas. Promoter methylation, gene copy number and mRNA expression of SIAH2 were determined in a panel of basal-like tumors and cell lines.

**Results:**

There was a significant increase in nuclear SIAH2 expression from normal breast tissues through to DCIS and progression to invasive cancers. A significant inverse correlation was apparent between SIAH2 and estrogen receptor and progesterone receptor and a positive association with tumor grade, HER2, p53 and an intrinsic basal-like subtype. Logistic regression analysis confirmed the significant positive association between SIAH2 expression and the basal-like phenotype. No SIAH2 promoter methylation was identified, yet there was a significant correlation between SIAH2 mRNA and gene copy number. SIAH2-positive tumors were associated with a shorter relapse-free survival in univariate but not multivariate analysis.

**Conclusions:**

SIAH2 expression is upregulated in basal-like breast cancers via copy number changes and/or transcriptional activation by p53 and is likely to be partly responsible for the enhanced hypoxic drive through abrogation of the prolyl hydroxylases.

## Introduction

Hypoxia in breast cancer has profound effects on tumor biology that are reflected in a poor prognosis and resistance to both chemotherapy and radiotherapy in patients [1]. Hypoxia-inducible factor (HIF)-1 is critical to the hypoxic response, being a transcription factor that, through binding to hypoxia response elements in the promoters of genes, results in expression of proteins involved in angiogenesis (vascular endothelial growth factor (VEGF)), glucose metabolism (glucose transporter 1), metastasis (chemokine (C-X-C motif) receptor 4 and stromal cell-derived factor-1), cell survival and proliferation.

HIF-1 is a dimer consisting of a constitutively expressed aryl nuclear translocator or HIF-1β and a hypoxia-inducible HIF-1α. The levels of HIF-1α are tightly regulated by three prolyl hydroxylases. In the presence of molecular oxygen, these enzymes hydroxylate the prolyl residues 402 and 564 in the oxygen-dependent domain of HIF-1α, resulting in conformational change and recognition by the von Hippel-Lindau protein that leads to its ubiquitination and degradation via the proteasome. In contrast, under hypoxia, the prolyl hydroxylases have limited molecular oxygen and are therefore less effective, which enables HIF-1α stabilization, translocation to the nucleus and initiation of gene transcription that benefits the tumor.

Seven *in absentia *homolog 2 (SIAH2) is one of a family of RING domain proteins which act alone or as components of ubiquitin ligase complexes that target proteins for proteasomal degradation [2]. Siah proteins can interact with many intracellular pathways, including the scaffold proteins, transcriptional repressors and nuclear receptor corepressors and β-catenin. Siah proteins are also involved in hypoxia signaling via regulation of HIF-1α [3] through the targeted degradation of prolyl hydroxylases under hypoxic conditions. Indeed, SIAH2-knockout mice have a delayed and abrogated response to hypoxic conditions that is mediated through reduced levels of HIF-1α [3,4]. These data suggest that Siah proteins may significantly alter HIF signaling through modulation of the prolyl hydroxylases.

Although the role of HIF has been documented in breast cancer [5,6], there are no data on the expression of SIAH2 in this disease. We have therefore investigated SIAH2 expression in breast cancer in two independent cohorts. Our aims were to (1) document the pattern and level of SIAH2 expression in breast cancer, (2) correlate expression with conventional clinicopathological factors, (3) investigate associations of SIAH2 expression with intrinsic subtypes of breast cancer and (4) determine the effect of SIAH2 expression on relapse-free survival.

## Materials and methods

### Patients

The flow of patients through the study according to the Reporting Recommendations for Tumor Marker Prognostic Studies (REMARK) criteria is listed in Supplementary Table 1 in Additional file 1[7] The first cohort was derived from the Department of Pathology, Peter MacCallum Cancer Centre, Melbourne, Australia, and comprised 120 cases with full clinicopathological characteristics but without survival data. The second cohort was from the Garvan Institute, Sydney, Australia, and comprised 293 cases with full clinicopathological characteristics including survival data [7]. In total, 439 invasive cancers with clinicopathological data and follow-up were available for study. Of these 439 cases, 61 cases were excluded because of inadequate tumor tissue on the array. The final cohort of invasive cancers comprised 378 cases (246 cases with survival data). Eighty cases of pure ductal carcinoma *in situ *(DCIS) were obtained from the John Radcliffe Hospital, Oxford, UK, of which 54 had DCIS on tissue microarrays (TMAs) for staining and clinical data available. Ten cases of normal postmenopausal breast tissues from mammoplasties were also collected. This study has Ethics Committee approvals (numbers 00/81, 03/90, 09/36, JRC02.216, HREC SVH H94/080 and H00/36). All patients had operable breast carcinomas and were not diagnosed with distant metastatic disease at the time of presentation. Information regarding patient characteristics, including age, tumor size, grade, histology and nodal status were collected from the clinical and pathological records. The median age of patients included in this study was 54 years (range, 24 to 87 years). Ninety-three percent of tumors were invasive ductal tumors not otherwise specified type, 3% were invasive lobular carcinomas and 4% were of other histological types (data were unavailable for two cases). Median tumor size was 20 mm, and the median tumor grade was 2. Forty-one percent of patients had nodal disease. Sixty-nine percent of tumors were estrogen receptor (ER)-positive, and 14% were human epidermal growth factor receptor 2 (HER2)-positive. Patients less than 50 years of age with node-positive, ER-negative tumors or tumors larger than 3 cm received adjuvant chemotherapy (cyclophosphamide, methotrexate and 5-fluorouracil or adriamycin and cyclophosphamide. Patients with hormone-responsive tumors who were more than 50 years of age received 5 years of endocrine therapy. Patients were followed up for a median period of 58.1 months. During this time, in the 100 patients from the invasive cohort, two developed recurrence and 86 deaths were considered breast cancer-related.

### Immunohistochemistry

TMAs were constructed from 1-mm diameter (invasive cancers) or 2-mm cores (DCIS). Sections of 4-μm thickness were used for immunostaining. TMA sections were dewaxed, and antigen retrieval was performed in 10 mM sodium citrate, pH 6, in a pressure cooker for 3 minutes. Sections were then treated with 3% H_2_O_2 _for 5 minutes to remove endogenous peroxides, washed and incubated with a SIAH2 antibody (NB110-88113; Novus Biologicals, Littleton, CO, USA) [9,10] at 1:50 dilution for 90 minutes at room temperature. The peroxidase-coupled Mouse ImmPRESS (Vector Laboratories, Burlingame, CA, USA) detection reagent was then used, and staining was visualized with diaminobenzidine plus (DAB+; Dako, Campbellfield, VIC, Australia). Sections were counterstained with hematoxylin to visualize nuclei. To analyze the expression of SIAH2 in breast cancer progression, we assessed expression using a combination of both intensity and proportion of cells expressing SIAH2 [10] Normal breast epithelium and tumors were scored for intensity (0 = no staining, 1 = weak staining, 2 = moderate staining and 3 = strong staining) and the percentage of cells (0 = no cells staining positive, 1 = <10% cells staining positive, 2 = 10% to 50% cells staining positive, 3 = 51% to 80% cells staining positive and 4 = >80% cells staining positive) as previously reported [12]. The scores for intensity and percentage of positive tumor cells were added to give a maximum score of 7. A cutoff of >2 (median value) was used to define two patient groups of approximately equal size for subsequent statistical analyses.

ER, HER2, epidermal growth factor receptor (EGFR) and cytokeratin (CK)5/6 staining were used to classify tumors into four intrinsic subgroups: the basal group (ER-negative, HER2-negative, CK5/6-positive and/or EGFR-positive), the luminal group (ER-positive, HER2-negative), the HER2 group (HER2-positive) and the negative (null) group (ER-negative, HER2-negative, CK5/6-negative and EGFR-negative) [13].

#### *Analysis of *SIAH2 *Methylation*

DNA from a separate series of 60 breast carcinomas (John Radcliffe Hospital, Oxford, UK) and five normal breast tissues (Peter MacCallum Cancer Centre, Melbourne, VIC, Australia), comprising all breast cancer phenotypes (50 ER-positive and 10 ER-negative) (also see Supplementary Table 2 in Additional file 2), and DNA was also obtained from the breast cancer cell lines MCF-10A, MCF-7, BT20, SkBr3, Hs578T, T47D, MDA-MB-157, MDA-MB-468, MDA-MB-453, MDA-MB-231, MDA-MB-361, BT483 and ZR75. Bisulfite-modified (EpiTect Bisulfite kit; Qiagen, Hilden, Germany) DNA were assessed for SIAH2 methylation using methylation-sensitive high-resolution melting (MS-HRM) [14]. The MS-HRM primers for SIAH2 were as follows: 5'-TAGAAGCGGGTGGGTTAGGGTTT-3' (forward) and 5'-CTAATACACTCCGCAACCCCC-3' (reverse) amplified a region corresponding to GenBank accession number AC011317.23, nucleotides 105279 to 105409, which contains 17 CpG islands. A polymerase chain reaction (PCR) assay was performed in a final volume of 20 μl. The PCR reaction mixture consisted of 1× PCR buffer (Qiagen), 2.5 mM MgCl_2_, 200 μM concentrations of each deoxyribonucleotide triphosphate, a 200 nM concentration of the forward primer, a 200 nM concentration of the reverse primer, 5 μM SYTO9 intercalating dye (Invitrogen, Mulgrave, VIC, Australia), 0.5 U of HotStarTaq DNA Polymerase (Qiagen), and 1 μl (theoretical amount 10 ng) of bisulfite-modified DNA. The PCR amplification was performed with an activation step of 15 minutes at 95°C, followed by 50 cycles of 10 seconds at 95°C, 10 seconds at an annealing temperature of 66°C, 20 seconds at 72°C for extension and one denaturation step of 1 minute at 97°C. HRM was directly performed after PCR amplification. PCR products were denatured at 97°C for 1 minute, then cooled to 75°C with temperature rising by 0.2°C per second to 97°C and holding for 1 second after each stepwise increment. Methylated sequences could be identified by their increased melting temperature [13]. In each assay, fully methylated, peripheral blood DNA (unmethylated), different methylation percentage dilution standards and nontemplate controls were included as controls and standards. All assays were performed in duplicate.

### Copy number analysis of SIAH2

A previous study analyzed both gene expression and copy number variation using the Illumina Human-6 BeadArray (Illumina, San Diego, CA, 92121, USA) and the CNV370 SNP array (Illumina) respectively, in a cohort of familial tumors [14]. These familial tumors were known to be breast cancer 1, early onset gene (*BRCA1*), *BRCA2 *or non-*BRCA1 *and non-*BRCA2 *tumors, and in this previous study the familial tumors were classified into one of the breast tumor subtypes: basal-like, luminal A, luminal B, HER2-positive and normal-like. These data were used to determine the expression of SIAH2 and its copy number status in 15 basal-like tumors. The copy number of SIAH2 in each tumor was inferred from the average logR value of eight single-nucleotide polymorphisms (SNPs), which were within the SIAH2 open reading frame (*n *= 3) or in the sequence flanking the gene (*n *= 5).

## Results

### SIAH2 **expression in normal breast, *in situ *and invasive breast carcinomas**

**Figure 1:**
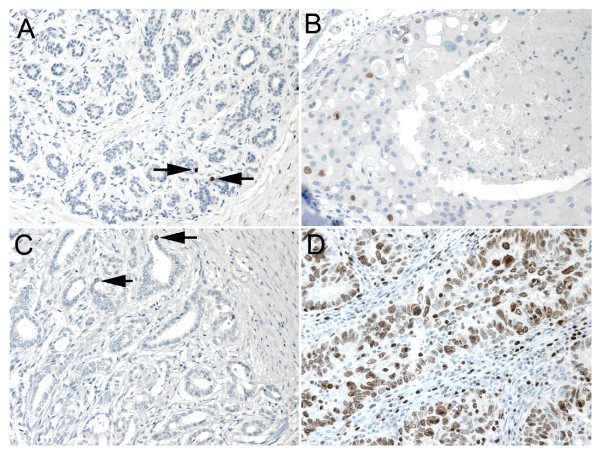
**Immunohistochemistry of seven *in absentia *homolog 2 (SIAH2) gene in normal, *in situ *and invasive breast carcinomas**. **(A) **Occasional nuclear positivity (arrows) in luminal cells in the terminal duct lobular unit. **(B) **Moderate to strong staining of SIAH2 in the nucleus of a small proportion of the cell in a high nuclear grade ductal carcinoma *in situ *with comedo necrosis. **(C) **Occasional weak to moderate SIAH2 (arrows) staining in a luminal type ductal carcinoma. **(D) **Strong SIAH2 staining in all nuclei in this basal-like breast carcinoma.

**Figure 2:**
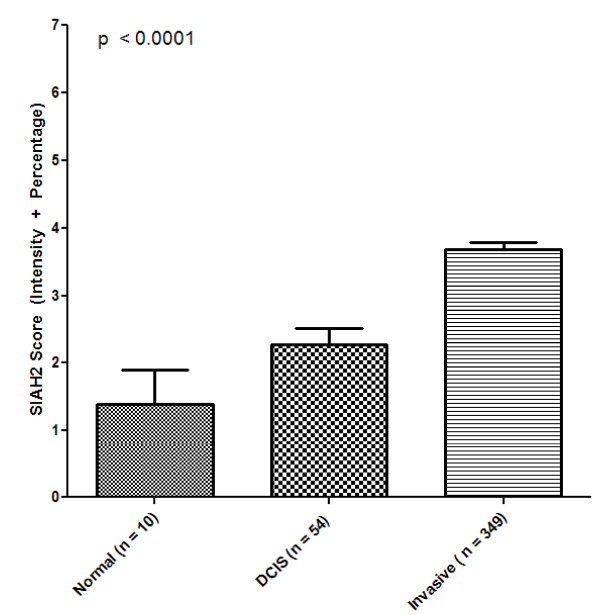
**Semiquantitative seven *in absentia *homolog 2 (SIAH2) gene expression in normal, ductal carcinoma *in situ *(DCIS) and invasive carcinoma samples**.

SIAH2 expression was identified in the nuclei of occasional cells within the luminal layer of ducts and acini in normal breast tissues in three of 10 patients (30%). This expression was usually of mild to moderate intensity, and when stratified using the cutoff used for the tumors, all were considered negative for SIAH2 (Figure 1A). Expression of SIAH2 was observed in the nuclei of seven of 54 (13%) DCIS cases. The staining was generally of a moderate to strong intensity with a homogeneous distribution (Figures 1B and 1C). There was a nonsignificant increase in SIAH2 from the transition of normal to *in situ *disease (*P *= 0.13) and a significant increase in SIAH2 from *in situ *to invasive breast carcinoma (*P *= 0.0006) (Table 1 and Figures 1D and 2).

**Table 1:** χ^2 ^tests, SIAH2 expression in normal breast, DCIS and invasive cancers^a^

Tissue type	Negative, *n *(%)	Positive, *n *(%)	Total, *n *(%)
Normal	10 (100%)	0 (0%)	10 (100%)
DCIS	47 (87%)	7 (13%)	54 (100%)
Invasive	155 (45%)	194 (55%)	349 (100%)
Total	212 (51%)	201 (49%)	413 (100%)

^**a**^SIAH2, seven *in absentia *homolog 2 gene; DCIS, ductal carcinoma *in situ*; *P *< 0.0001.

### Association between SIAH2 protein expression and clinicopathological characteristics in DCIS

There was no significant correlation between SIAH2 expression and nuclear grade, presence of necrosis, age, ER, progesterone receptor (PR), EGFR or HER2 (all *P *> 0.05) or intrinsic phenotypes in DCIS (*P *= 0.471) (Supplementary Table 3 in Additional file 3).

### Correlation between SIAH2 protein expression with clinicopathological characteristics and intrinsic subtypes in invasive cancer

In the primary cohort, there was a significant inverse correlation between SIAH2 protein expression and ER (*P *< 0.0001), PR (*P *= 0.011) and a positive association with tumor grade (*P *< 0.0001) and intrinsic subtype (*P *= 0.028), but there was no association with patient age, tumor size, lymph node status or HER2 (all *P *> 0.05) (Supplementary Table 4 in Additional file 4). In the validation cohort, there was a significant inverse correlation between SIAH2, ER (*P *< 0.0001) and PR (*P *< 0.0001) and a significant positive association with tumor grade (*P *< 0.0001), patient age (*P *= 0.009), HER2 (*P *= 0.007) and intrinsic subtype (*P *< 0.0001), but not with tumor size or lymph node status (*P *> 0.05) (Supplementary Table 5 in Additional file 5). In the combined cohort, there was a significant inverse correlation between SIAH2 and ER (*P *< 0.0001) and PR (*P *< 0.0001) and a positive correlation with tumor grade (*P *< 0.0001), HER2 (*P *= 0.007), p53 (*P *< 0.001) and intrinsic subtype (*P *< 0.0001), but not with patient age, tumor size or lymph node status (*P *> 0.05) (Table 2). The significant associations between SIAH2 expression grade and intrinsic subgroups were confirmed in a multivariate analysis of tumor phenotype, age, grade and lymph node status with the basal-like phenotype being more than five times more likely to express SIAH2 than luminal tumors (Table 3) (*P *= 0.015), which was also observed in the combined cohort (*P *= 0.042).

**Table 2:** Contingency table of SIAH2 expression in invasive breast carcinomas of the combined cohort with clinicopathological parameters

Parameter	Negative, *N *= 169 (45%)	Positive, *N *= 209 (55%)	Total, *N *= 378 (100%)	*P *value
Grade				<0.0001
Low	40 (78.4%)	11 (21.6%)	51 (100%)	
Intermediate	77 (60.2%)	51 (39.8%)	128 (100%)	
High	32 (20.4%)	125 (79.6%)	157 (100%)	
Age, yr				0.187
<50	48 (39.3%)	74 (60.7%)	122 (100%)	
>50	107 (47.8%)	117 (52.1%)	224 (100%)	
Tumor size				0.168
<20 mm	89 (48.6%)	94 (51.4%)	183 (100%)	
>20 mm	61 (39.9%)	92 (60.1%)	153 (100%)	
Lymph node status				0.915
Negative	83 (45.4%)	100 (54.6%)	147 (100%)	
Positive	64 (43.0%)	85 (57.0%)	185 (100%)	
ER status				<0.0001
Negative	21 (20.8%)	80 (79.2%)	101 (100%)	
Positive	129 (54.4%)	108 (45.6%)	237 (100%)	
PR status				<0.0001
Negative	39 (28.5%)	98 (71.5%)	137 (100%)	
Positive	111 (55.2%)	90 (44.8%)	201 (100%)	
HER2 status				0.003
Negative	128 (48.3%)	137 (51.7%)	265 (100%)	
Positive	22 (29.7%)	52 (70.3%)	74 (100%)	
Tumor subtype				<0.0001
Luminal	114 (57.6%)	84 (42.4%)	198 (100%)	
Basal-like	4 (9.6%)	37 (90.2%)	41 (100%)	
Her2	22 (29.3%)	53 (70.7%)	75 (100%)	
Null	11 (44%)	14 (56%)	25 (100%)	
P53				
Negative	108 (57.8%)	79 (42.2%)	187 (100%)	<0.001
Positive	12 (18.2%)	54 (81.8%)	66 (100%)	

^a^SIAH2, seven *in absentia *homolog 2 gene.

ER, estrogen receptor; HER2, human epidermal growth factor receptor 2; PR, progesterone receptor.

**Table 3:** Multivariate analysis in the combined cohort (*N *= 378)

Characteristic	*P *value	Hazard ratio	95% CI for hazard ratios
Tumor type			
Luminal (reference)	0.09		
Basal	0.02	4.04	1.3 to 12.8
HER2	0.15	1.64	0.8 to 3.2
Null	0.67	1.23	0.5 to 3.2
Grade			
1	0.000		
2	0.02	2.61	1.2 to 5.8
3	0.0001	12.72	5.2 to 31.4
Lymph node status	0.28	0.75	0.4 to 1.3
Tumor size >20 mm	0.18	0.69	0.4 to 1.2

Multivariate analysis using a binary logistic regression model of the effect of SIAH2 expression on tumor subtype (with luminal tumors as a reference), tumor grade, lymph node status and tumor size

^aa^SIAH2, seven *in absentia *homolog 2 gene.

95% CI, 95% confidence interval; HER2, human epidermal growth factor receptor 2.

### Promoter methylation of SIAH2 in cell lines and tumors

To assess whether SIAH2 expression in tumors is modulated by promoter methylation, CpG islands were identified in the promoter region of SIAH2, and MS-HRM primers were designed to cover the CpG-rich area of the promoter region of SIAH2. Five normal breast tissues, 60 breast carcinomas and 13 breast cancer cell lines were screened for methylation of SIAH2, but no promoter methylation was detected in these cancer cell lines and samples, as shown by the absence of altered methylation profiles (Supplementary Figure 1 in Additional file 6).

### Correlation of gene expression and relative copy number of SIAH2 in basal-like tumors

**Figure 3:**
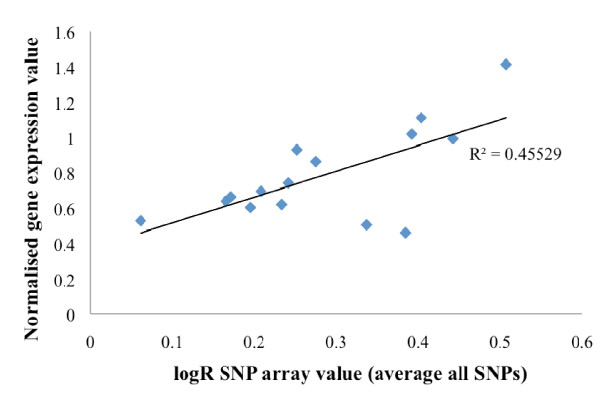
**Correlation between seven *in absentia *homolog 2 (SIAH2) gene copy number changes as assessed by the average logR array value of eight single-nucleotide polymorphisms (SNPs) which located in SIAH2 or within the flanking region of the gene and normalized expression of SIAH2 in 15 basal-like breast cancers**.

A cohort of familial tumors, which included 15 basal-like tumors, was previously analyzed on the basis of gene expression and copy number analysis [15]. The 15 basal-like tumors showed a significant correlation between SIAH2 expression and estimated copy number (*r *= 0.675, *P *= 0.003) (Figure 3). Two of the 15 basal-like tumors showed a copy number gain (copy number of 3) and a further three of 15 basal-like tumors showed loss of heterozygosity at this region. In contrast, 15 non-basal-like tumors (10 luminal A, four luminal B and one normal-like tumor) did not show any copy number change in this region.

### Relationship between SIAH2 expression and relapse-free and overall survival

**Figure 4:**
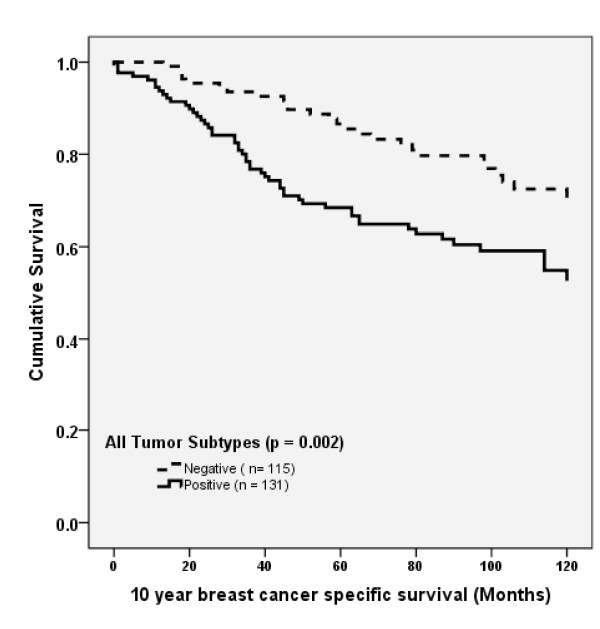
**Kaplan-Meier curves stratified by seven *in absentia *homolog 2 (SIAH2) gene expression for relapse-free survival in (A) all tumors (*N *= 246) and (B) luminal tumors (*n *= 144)**.

There was no correlation present between SIAH2 expression and overall relapse-free survival in DCIS-only patients (*P *= 0.68). Although there was a significantly shorter relapse-free survival in all patients with invasive carcinomas stratified by SIAH2 (*P *= 0.002) (Figure 4), no significant association with relapse-free survival was observed in univariate analysis in different breast cancer intrinsic groups stratified by SIAH2 (data not shown). There was also no significant association between SIAH2 in invasive carcinomas of all patients and relapse-free survival in multivariate analysis (Table 4).

**Table 4:** Multivariate analysis using the Cox regression model of relapse-free survival in all breast cancers of validation cohort (*N *= 245)^a^

Characteristic	*P *value	Hazard ratio	95% CI for hazard ratios
SIAH2	0.47	1.24	0.7 to 2.2
Grade			
1 (reference)	0.54		
2	0.70	0.83	0.3 to 2.1
3	0.70	1.22	0.4 to 3.3
Tumor size	0.12	1.37	0.9 to 2.2
Lymph node status	0.001	2.25	1.4 to 3.7
Tumor type			
Luminal	0.004		
Basal-like	0.005	3.0	1.4 to 6.4
HER2	0.001	2.9	1.6 to 5.7
Null	0.016	2.5	1.2 to 5.1

^a^SIAH2, seven *in absentia *homolog 2 gene.

95% CI, 95% confidence interval, HER2, human epidermal growth factor receptor 2.

## Discussion

Hypoxia is a pivotal driver in breast tumor progression, leading to transcription of several suites of genes involved in angiogenesis, cell survival, cell proliferation and an enhanced metastatic phenotype that are advantageous to the neoplastic cells [1]. SIAH2 is part of the ubiquitin ligase complex that target proteins for proteasomal degradation and enhances HIF-1α expression by reducing the abundance of the prolyl hydroxylases [16,17]. These enzymes, in the absence of SIAH2, hydroxylate prolyl residues in the oxygen-dependent domain of HIF-1α, resulting in HIF-1α proteasomal degradation and attenuation of the hypoxic response. Since SIAH2 has the potential to profoundly influence the hypoxic response, we investigated its expression in normal and neoplastic breast tissues.

We observed significant upregulation of SIAH2 in the nucleus in the transition from normal to *in situ *and invasive carcinomas in breast cancer, supporting the notion of an important role of SIAH2 in breast cancer progression. SIAH2 has a nuclear localization signal that could account for its subcellular pattern of expression [18]. The increase in SIAH2 in *in situ *and invasive carcinomas correlates with the hypoxia that occurs in neoplasia as the metabolic demand of the tumor exceeds the supply of nutrients and oxygen from the disordered vasculature that is developing.

Correlation analysis showed a significant relationship between high levels of breast tumor SIAH2, negative ER and PR and high HER2. The absence of a positive correlation with ER is of interest, since estrogen has been reported to induce expression of SIAH2 in ER-positive breast cancer cell lines [19] and there is a positive relationship between Siah2 and ER-positive breast tumors but not the basal-like phenotype in six publicly accessible data sets [7,20-25] (analysis not shown). This discrepancy is likely due to the well-described differences between gene expression and protein abundance or to the fact that ER expression may be heterogeneous in both the pattern and level of expression within tumors. Nevertheless, although there was an inverse relationship between SIAH2 and ER, approximately half of SIAH2-positive samples expressed ER, suggesting a complex relationship.

The finding that SIAH2 was significantly associated with HER2 and basal-like intrinsic breast cancer subtypes, which for basal-like cancers was confirmed in multivariate analysis, is in accord with our previous report of an enhanced hypoxic drive in basal-like cancers [26]. In this study, we have demonstrated that basal-like breast cancers have an intrinsically elevated SIAH2 level as part of its phenotype that may, partly at least, explain the mechanism underlying high HIF-1α expression in this tumor subtype. The upregulation of SIAH2 may be regulated at several levels. We investigated the potential role that *p53 *may play, since this gene is frequently mutated in this tumor type [11]. In support of this notion is the significant correlation between SIAH2 and p53 immunostaining. A further mechanism in basal-like cancer may also involve p38 mitogen-activated protein kinase, which is also upregulated in the basal-like phenotype, as activated p38 increases the activity of SIAH2 [11]. We also explored the role of SIAH2 promoter methylation to assess whether protein expression is epigenetically repressed. We observed no evidence of methylation in any breast carcinoma cell line, normal breast or in a series of 60 breast cancers of variable phenotypes, making this mechanism of repression highly unlikely in breast tissues in either normal tissue or tumoral tissue.

We then hypothesized that since SIAH2 is located on 3q25.1, overexpression might be mediated through gene amplification. Indeed, this locus is frequently amplified in basal-like breast cancer [27], and our preliminary results showed a significant correlation between DNA copy number and mRNA expression, supporting this hypothesis. Specifically, we found that basal-like tumors showed copy number gain of the SIAH2 locus more frequently than luminal tumors and that basal-like tumors containing copy number gain were associated with high expression of SIAH2. Nevertheless, using a more sensitive and specific method for quantifying gene copy number such as fluorescence *in situ *hybridization assay, together with SIAH2 protein expression in a validation cohort, would be of interest to confirm this finding and assess whether true amplification occurs.

Although we observed a significantly shorter relapse-free survival in patients with SIAH2-positive tumors in univariate analysis, this was not confirmed in the multivariate analysis model that included conventional prognostic factors such as tumor size, tumor grade and lymph node status. While SIAH2 was not an independent survival factor in a multivariate analysis model, it was prognostic in the univariate analysis because of its strong association with the basal-like phenotype, which is an independent prognostic factor. Even so, the role of SIAH2 remains unclear. Thus a report has suggested that patients with SIAH2-positive, ER-positive tumors have a significantly longer progression-free survival than patients with SIAH2-negative tumors and also that Siah2 levels might be a predictive marker of estrogen-responsive disease [28]. The discrepancy between these findings and our own are likely due to the use of mRNA levels to measure SIAH2 by Jansen *et al. *[28], and, despite enriching for neoplastic cells, the stromal compartment contributed to the overexpression of SIAH2, thus confounding the comparison. In support of this notion, it has been shown by expression microarrays that downregulated SIAH2 in brain metastasis of breast cancer corresponds with low stromal contamination [29]. The concept of SIAH2 being a good prognostic and/or predictive parameter is not in accord with the role of SIAH2 in regulating the hypoxic response or with the observation that inhibition of SIAH2 is associated with reduced metastases in animal models [30]. SIAH2 appears to have several mechanisms of mediating its effect. Some SIAH2 substrates bind directly through an AXVXP motif, some require adaptor proteins and still others are targeted independently of the above sequence motif [3]. Thus, depending on the cell context, SIAH2 is likely to have a variety of effects.

SIAH2 is critical to the level of the hypoxic response and therefore is a potential target for anticancer therapy. Indeed, since HIF activation results in the regulation of a large number of genes, interference with this pathway would have broad antineoplastic effects in contrast to targeting individual genes, such as VEGF, with bevacizumab, which is currently used in the clinic. Indeed, menadione, a specific inhibitor of SIAH2, increased expression of prolyl hydroxylase with a concomitant decrease in levels of HIF-1α. This promising therapeutic approach also retarded the growth of melanoma xenografts [31]. The potential of this approach has also been investigated using a short protein fragment that competitively binds to Siah, resulting in reduced breast cancer growth, which appeared to be mediated through inhibition of the hypoxic response [4].

## Conclusions

In summary, we have shown that *in situ *and invasive breast carcinomas upregulate SIAH2 and that it is preferentially highly expressed in the basal-like subtype, which can be accounted for in part by increased gene copy number. High levels of SIAH2 may be partly responsible for the enhanced hypoxic drive that underlies this tumor type, which is chemotherapy- and radiotherapy-resistant. Targeting SIAH2, the most apical regulator identified in the hypoxic response pathway, may be a suitable option for anticancer therapy in this breast tumor subtype.

## Abbreviations

ER: estrogen receptor; HIF: hypoxia-inducible factor; PR: progesterone receptor; Siah: seven *in absentia *homologue.

## Competing interests

The authors declare that they have no competing interests.

## Authors' contributions

PC carried out the scoring of the tissue microarrays (TMAs) and calculated statistics. AM conceived and designed the study and drafted the manuscript. MCPL and JES stained the TMAs. CSFW assisted in staining the TMAs and drafting the manuscript. NW conducted copy number experiments and analysis. KTH conducted the promoter analysis. AD conducted and supervised promoter analysis. EKAM and SAO generated TMAs and scored correlative markers. CMM conducted clinical follow-up. RLS provided TMAs, helped in drafting the manuscript and provided critical discussions. DB helped in designing the study. SBF scored the TMAs, conceived and designed the study and drafted the manuscript. All authors read and approved the final version of the manuscript.

## Supplementary Material

Additional file 1 **Supplementary Table 1**. Flow of breast cancer patients through the study, according to REMARK criteria [7] Click here for file

Additional file 2 **Supplementary Table 2**. Tumor phenotype of 60 samples for which seven *in absentia *homolog 2 (SIAH2) gene methylation analysis was performed Click here for file

Additional file 3 **Supplementary Table 3**. Contingency table of ductal carcinoma *in situ *(DCIS) and available clinicopathological variables Click here for file

Additional file 4 **Supplementary Table 4**. Contingency table of seven *in absentia *homolog 2 (SIAH2) gene expression in invasive breast carcinomas of the primary cohort with clinicopathological parameters Click here for file

Additional file 5 **Supplementary Table 5**. Contingency table of seven *in absentia *homolog 2 (SIAH2) gene expression in invasive breast carcinomas of the initial cohort with clinicopathological parameters Click here for file

Additional file 6 **Supplementary Figure 1**. Seven *in absentia *homolog 2 (SIAH2) gene methylation in breast carcinoma samples. Methylation-sensitive high-resolution melting (MS-HRM) detects sample methylation status by melting the amplicons after polymerase chain reaction assay. Methylated samples melt later than unmethylated samples, as they have cytosines in their sequences rather than the thymines after the bisulfite modification. Two estrogen receptor (ER)-positive and two ER-negative breast carcinomas show no methylation in SIAH2. Standard controls of 100%, 50%, 10% and 0% methylation are shown. The curve for each sample represents data from duplicate samples. Click here for file

## References

[B1] Harris AL Hypoxia: a key regulatory factor in tumour growth Nat Rev Cancer 2002 2 38 47 10.1038/nrc704 11902584

[B2] House CM Moller A Bowtell DD Siah proteins: novel drug targets in the Ras and hypoxia pathways Cancer Res 2009 69 8835 8838 10.1158/0008-5472.CAN-09-1676 19920190

[B3] Nakayama K Qi J Ronai Z The ubiquitin ligase Siah2 and the hypoxia response Mol Cancer Res 2009 7 443 451 10.1158/1541-7786.MCR-08-0458 19372575 PMC2860273

[B4] Moller A House CM Wong CS Scanlon DB Liu MC Ronai Z Bowtell DD Inhibition of Siah ubiquitin ligase function Oncogene 2009 28 289 296 10.1038/onc.2008.382 18850011 PMC3000903

[B5] Bos R Zhong H Hanrahan CF Mommers EC Semenza GL Pinedo HM Abeloff MD Simons JW van Diest PJ van der Wall E Levels of hypoxia-inducible factor-1α during breast carcinogenesis J Natl Cancer Inst 2001 93 309 314 10.1093/jnci/93.4.309 11181778

[B6] Dales JP Garcia S Meunier-Carpentier S Andrac-Meyer L Haddad O Lavaut MN Allasia C Bonnier P Charpin C Overexpression of hypoxia-inducible factor HIF-1α predicts early relapse in breast cancer: retrospective study in a series of 745 patients Int J Cancer 2005 116 734 739 10.1002/ijc.20984 15849727

[B7] Tan EY Yan M Campo L Han C Takano E Turley H Candiloro I Pezzella F Gatter KC Millar EK O'Toole SA McNeil CM Crea P Segara D Sutherland RL Harris AL Fox SB The key hypoxia regulated gene *CAIX *is upregulated in basal-like breast tumours and is associated with resistance to chemotherapy Br J Cancer 2009 100 405 411 10.1038/sj.bjc.6604844 19165203 PMC2634728

[B8] Millar EK Anderson LR McNeil CM O'Toole SA Pinese M Crea P Morey AL Biankin AV Henshall SM Musgrove EA Sutherland RL Butt AJ BAG-1 predicts patient outcome and tamoxifen responsiveness in ER-positive invasive ductal carcinoma of the breast Br J Cancer 2009 100 123 133 10.1038/sj.bjc.6604809 19066611 PMC2634679

[B9] Schmidt RL Park CH Ahmed AU Gundelach JH Reed NR Cheng S Knudsen BE Tang AH Inhibition of RAS-mediated transformation and tumorigenesis by targeting the downstream E3 ubiquitin ligase seven in absentia homologue Cancer Res 2007 67 11798 11810 10.1158/0008-5472.CAN-06-4471 18089810

[B10] Ahmed AU Schmidt RL Park CH Reed NR Hesse SE Thomas CF Molina JR Deschamps C Yang P Aubry MC Tang AH Effect of disrupting seven-in-absentia homolog 2 function on lung cancer cell growth J Natl Cancer Inst 2008 100 1606 1629 10.1093/jnci/djn365 19001609 PMC2720765

[B11] Tan EY Campo L Han C Turley H Pezzella F Gatter KC Harris AL Fox SB Cytoplasmic location of factor-inhibiting hypoxia-inducible factor is associated with an enhanced hypoxic response and a shorter survival in invasive breast cancer Breast Cancer Res 2007 9 R89 10.1186/bcr1838 18096060 PMC2246192

[B12] Mirzoeva OK Das D Heiser LM Bhattacharya S Siwak D Gendelman R Bayani N Wang NJ Neve RM Guan Y Hu Z Knight Z Feiler HS Gascard P Parvin B Spellman PT Shokat KM Wyrobek AJ Bissell MJ McCormick F Kuo WL Mills GB Gray JW Korn WM Basal subtype and MAPK/ERK kinase (MEK)-phosphoinositide 3-kinase feedback signaling determine susceptibility of breast cancer cells to MEK inhibition Cancer Res 2009 69 565 572 10.1158/0008-5472.CAN-08-3389 19147570 PMC2737189

[B13] Nielsen TO Hsu FD Jensen K Cheang M Karaca G Hu Z Hernandez-Boussard T Livasy C Cowan D Dressler L Akslen LA Ragaz J Gown AM Gilks CB van de Rijn M Perou CM Immunohistochemical and clinical characterization of the basal-like subtype of invasive breast carcinoma Clin Cancer Res 2004 10 5367 5374 10.1158/1078-0432.CCR-04-0220 15328174

[B14] Wojdacz TK Dobrovic A Methylation-sensitive high resolution melting (MS-HRM): a new approach for sensitive and high-throughput assessment of methylation Nucleic Acids Res 2007 35 e41 10.1093/nar/gkm013 17289753 PMC1874596

[B15] Waddell N Arnold J Cocciardi S da Silva L Marsh A Riley J Johnstone CN Orloff M Assie G Eng C Reid L Keith P Yan M Fox S Devilee P Godwin AK Hogervorst FB Couch F Investigators kConFab Grimmond S Flanagan JM Khanna K Simpson PT Lakhani SR Chenevix-Trench G Subtypes of familial breast tumours revealed by expression and copy number profiling Breast Cancer Res Treat 2010 123 661 677 10.1007/s10549-009-0653-1 19960244

[B16] Fukuba H Yamashita H Nagano Y Jin HG Hiji M Ohtsuki T Takahashi T Kohriyama T Matsumoto M Siah-1 facilitates ubiquitination and degradation of factor inhibiting HIF-1α (FIH) Biochem Biophys Res Commun 2007 353 324 329 10.1016/j.bbrc.2006.12.051 17188242

[B17] Simon MC Siah proteins, HIF prolyl hydroxylases, and the physiological response to hypoxia Cell 2004 117 851 853 10.1016/j.cell.2004.06.010 15210106

[B18] Della NG Senior PV Bowtell DD Isolation and characterisation of murine homologues of the *Drosophila **seven in absentia *gene (*sina*) Development 1993 117 1333 1343 8404535 10.1242/dev.117.4.1333

[B19] Frasor J Danes JM Funk CC Katzenellenbogen BS Estrogen down-regulation of the corepressor N-CoR: mechanism and implications for estrogen derepression of N-CoR-regulated genes Proc Natl Acad Sci USA 2005 102 13153 13157 10.1073/pnas.0502782102 16141343 PMC1201577

[B20] Chin K DeVries S Fridlyand J Spellman PT Roydasgupta R Kuo WL Lapuk A Neve RM Qian Z Ryder T Chen F Feiler H Tokuyasu T Kingsley C Dairkee S Meng Z Chew K Pinkel D Jain A Ljung BM Esserman L Albertson DG Waldman FM Gray JW Genomic and transcriptional aberrations linked to breast cancer pathophysiologies Cancer Cell 2006 10 529 541 10.1016/j.ccr.2006.10.009 17157792

[B21] Desmedt C Piette F Loi S Wang Y Lallemand F Haibe-Kains B Viale G Delorenzi M Zhang Y d'Assignies MS Bergh J Lidereau R Ellis P Harris AL Klijn JG Foekens JA Cardoso F Piccart MJ Buyse M Sotiriou C Strong time dependence of the 76-gene prognostic signature for node-negative breast cancer patients in the TRANSBIG multicenter independent validation series Clin Cancer Res 2007 13 3207 3214 10.1158/1078-0432.CCR-06-2765 17545524

[B22] Hess KR Anderson K Symmans WF Valero V Ibrahim N Mejia JA Booser D Theriault RL Buzdar AU Dempsey PJ Rouzier R Sneige N Ross JS Vidaurre T Gomez HL Hortobagyi GN Pusztai L Pharmacogenomic predictor of sensitivity to preoperative chemotherapy with paclitaxel and fluorouracil, doxorubicin, and cyclophosphamide in breast cancer J Clin Oncol 2006 24 4236 4244 10.1200/JCO.2006.05.6861 16896004

[B23] Miller LD Smeds J George J Vega VB Vergara L Ploner A Pawitan Y Hall P Klaar S Liu ET Bergh J An expression signature for p53 status in human breast cancer predicts mutation status, transcriptional effects, and patient survival Proc Natl Acad Sci USA 2005 102 13550 13555 10.1073/pnas.0506230102 16141321 PMC1197273

[B24] Minn AJ Gupta GP Siegel PM Bos PD Shu W Giri DD Viale A Olshen AB Gerald WL Massague J Genes that mediate breast cancer metastasis to lung Nature 2005 436 518 524 10.1038/nature03799 16049480 PMC1283098

[B25] Wang Y Klijn JG Zhang Y Sieuwerts AM Look MP Yang F Talantov D Timmermans M Meijer-van Gelder ME Yu J Jatkoe T Berns EM Atkins D Foekens JA Gene-expression profiles to predict distant metastasis of lymph-node-negative primary breast cancer Lancet 2005 365 671 679 15721472 10.1016/S0140-6736(05)17947-1

[B26] Manié E Vincent-Salomon A Lehmann-Che J Pierron G Turpin E Warcoin M Gruel N Lebigot I Sastre-Garau X Lidereau R Remenieras A Feunteun J Delattre O de Thé H Stoppa-Lyonnet D Stern MH High frequency of *TP53 *mutation in *BRCA1 *and sporadic basal-like carcinomas but not in *BRCA1 *luminal breast tumors Cancer Res 2009 69 663 671 19147582 10.1158/0008-5472.CAN-08-1560

[B27] Gatza ML Lucas JE Barry WT Kim JW Wang Q Crawford MD Datto MB Kelley M Mathey-Prevot B Potti A Nevins JR A pathway-based classification of human breast cancer Proc Natl Acad Sci USA 2010 107 6994 6999 10.1073/pnas.0912708107 20335537 PMC2872436

[B28] Jansen MP Ruigrok-Ritstier K Dorssers LC van Staveren IL Look MP Meijer-van Gelder ME Sieuwerts AM Helleman J Sleijfer S Klijn JG Foekens JA Berns EM Downregulation of SIAH2, an ubiquitin E3 ligase, is associated with resistance to endocrine therapy in breast cancer Breast Cancer Res Treat 2009 116 263 271 10.1007/s10549-008-0125-z 18629630

[B29] Palmieri D Fitzgerald D Shreeve SM Hua E Bronder JL Weil RJ Davis S Stark AM Merino MJ Kurek R Mehdorn HM Davis G Steinberg SM Meltzer PS Aldape K Steeg PS Analyses of resected human brain metastases of breast cancer reveal the association between up-regulation of hexokinase 2 and poor prognosis Mol Cancer Res 2009 7 1438 1445 10.1158/1541-7786.MCR-09-0234 19723875 PMC2746883

[B30] Qi J Nakayama K Gaitonde S Goydos JS Krajewski S Eroshkin A Bar-Sagi D Bowtell D Ronai Z The ubiquitin ligase Siah2 regulates tumorigenesis and metastasis by HIF-dependent and -independent pathways Proc Natl Acad Sci USA 2008 105 16713 16718 10.1073/pnas.0804063105 18946040 PMC2575485

[B31] Shah M Stebbins JL Dewing A Qi J Pellecchia M Ronai ZA Inhibition of Siah2 ubiquitin ligase by vitamin K_3 _(menadione) attenuates hypoxia and MAPK signaling and blocks melanoma tumorigenesis Pigment Cell Melanoma Res 2009 22 799 808 10.1111/j.1755-148X.2009.00628.x 19712206 PMC2863310

