# Supplementary material for: The expression of the ubiquitin ligase SIAH2 (seven in absentia homolog 2) is mediated through gene copy number in breast cancer and is associated with a basal-like phenotype and p53 expression
Source: Breast Cancer Res. 2011 Feb 9;13(1):R19. doi: 10.1186/bcr2828 (PMC3109588; doi:10.1186/bcr2828)
Supplement: Additional file 1 — Supplementary Table 1. Flow of breast cancer patients through the study, according to REMARK criteria [7] [file bcr2828-S1.PPT]

## Slide 1
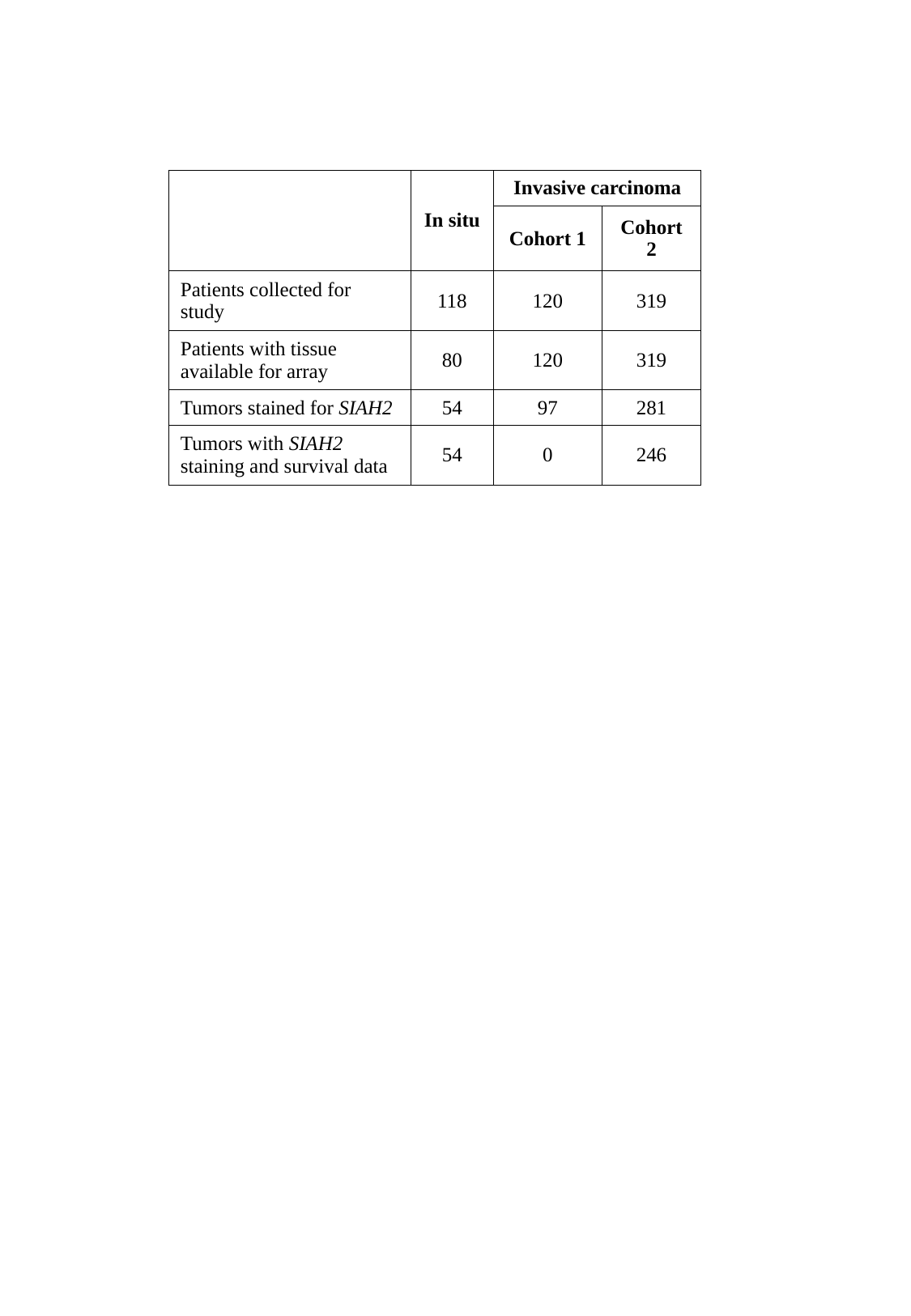

| | In situ | Invasive carcinoma | |
| --- | --- | --- | --- |
| | | Cohort 1 | Cohort 2 |
| Patients collected for study | 118 | 120 | 319 |
| Patients with tissue available for array | 80 | 120 | 319 |
| Tumors stained for SIAH2 | 54 | 97 | 281 |
| Tumors with SIAH2 staining and survival data | 54 | 0 | 246 |
