# Supplementary figures and images for: The expression of the ubiquitin ligase SIAH2 (seven in absentia homolog 2) is mediated through gene copy number in breast cancer and is associated with a basal-like phenotype and p53 expression
Source: Breast Cancer Res. 2011 Feb 9;13(1):R19. doi: 10.1186/bcr2828 (PMC3109588; doi:10.1186/bcr2828)

## Slide 1
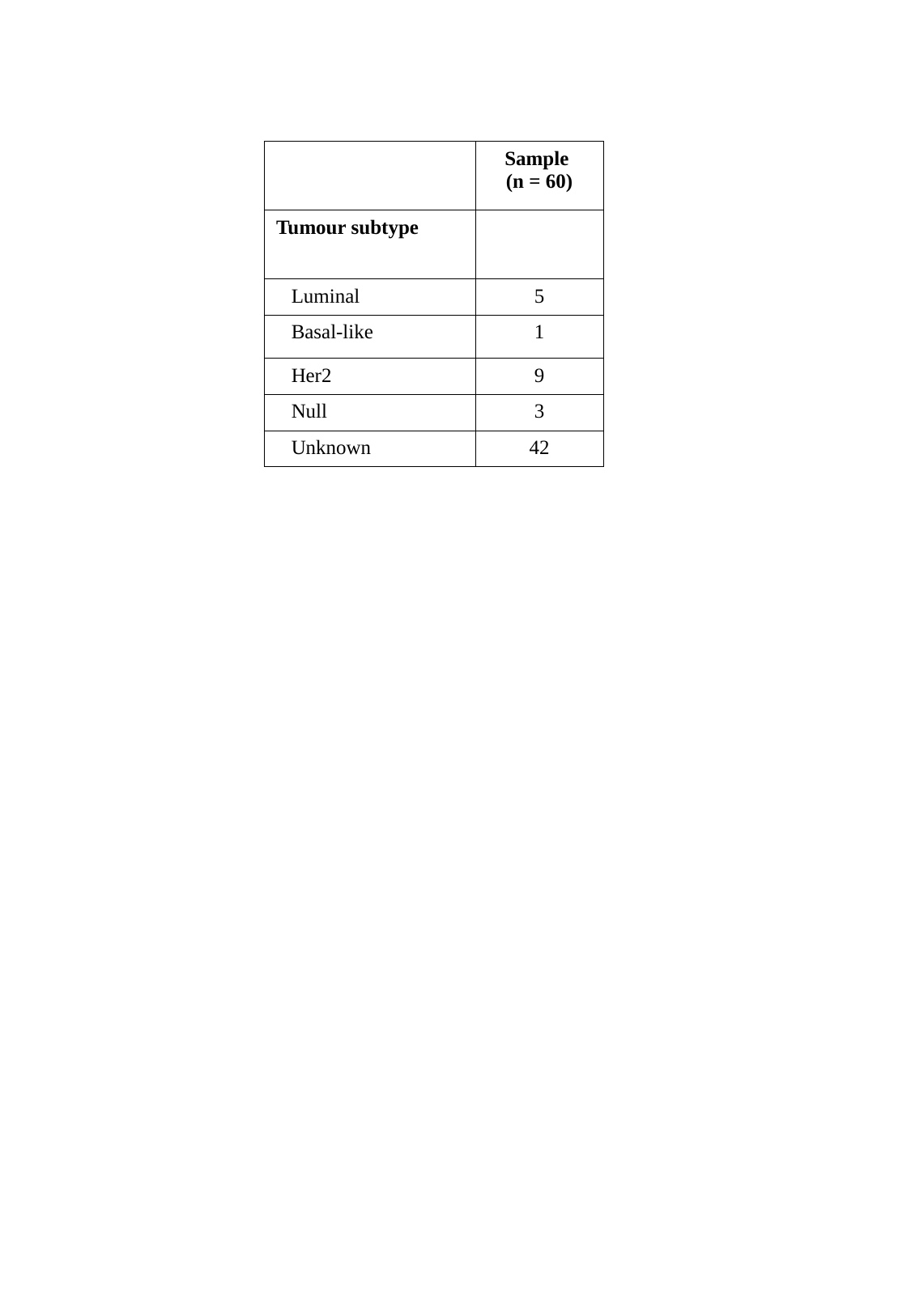

| | Sample (n = 60) |
| --- | --- |
| Tumour subtype | |
| Luminal | 5 |
| Basal-like | 1 |
| Her2 | 9 |
| Null | 3 |
| Unknown | 42 |

Supplement: Additional file 2 — Supplementary Table 2. Tumor phenotype of 60 samples for which seven in absentia homolog 2 (SIAH2) gene methylation analysis was performed [file bcr2828-S2.PPT]

## Slide 1
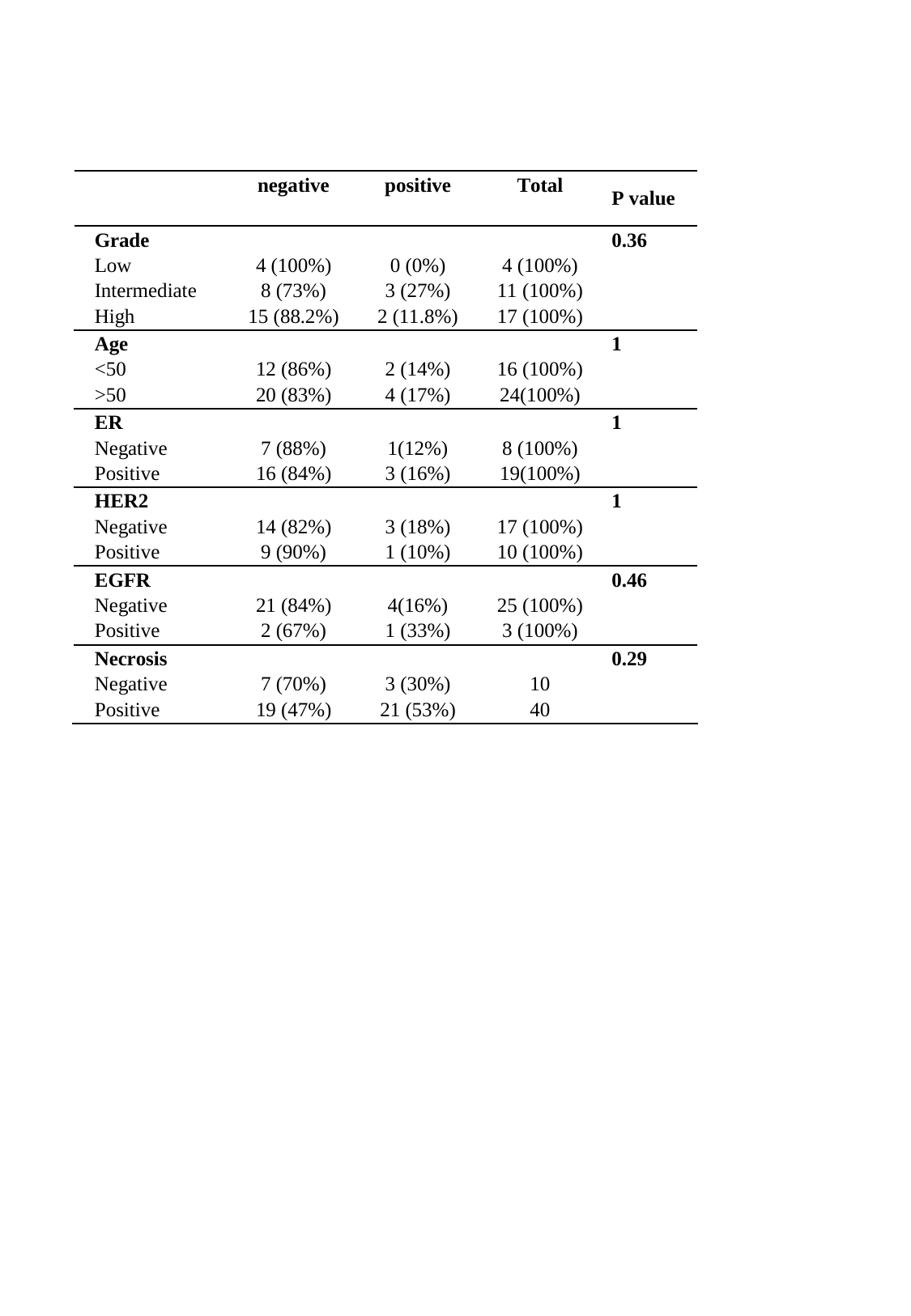

Supplement: Additional file 3 — Supplementary Table 3. Contingency table of ductal carcinoma in situ (DCIS) and available clinicopathological variables [file bcr2828-S3.PPT]

## Slide 1
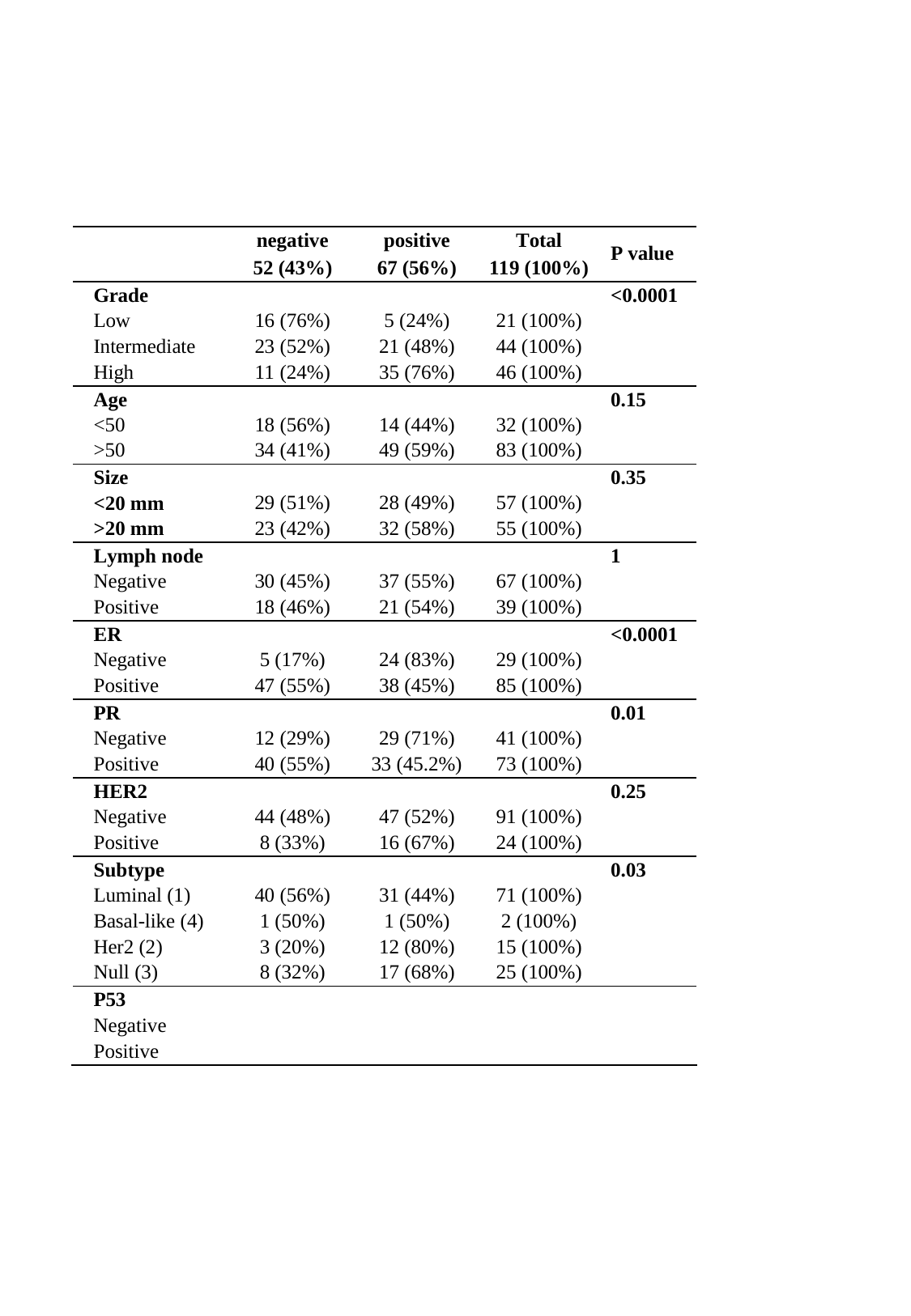

Supplement: Additional file 4 — Supplementary Table 4. Contingency table of seven in absentia homolog 2 (SIAH2) gene expression in invasive breast carcinomas of the primary cohort with clinicopathological parameters [file bcr2828-S4.PPT]

## Slide 1
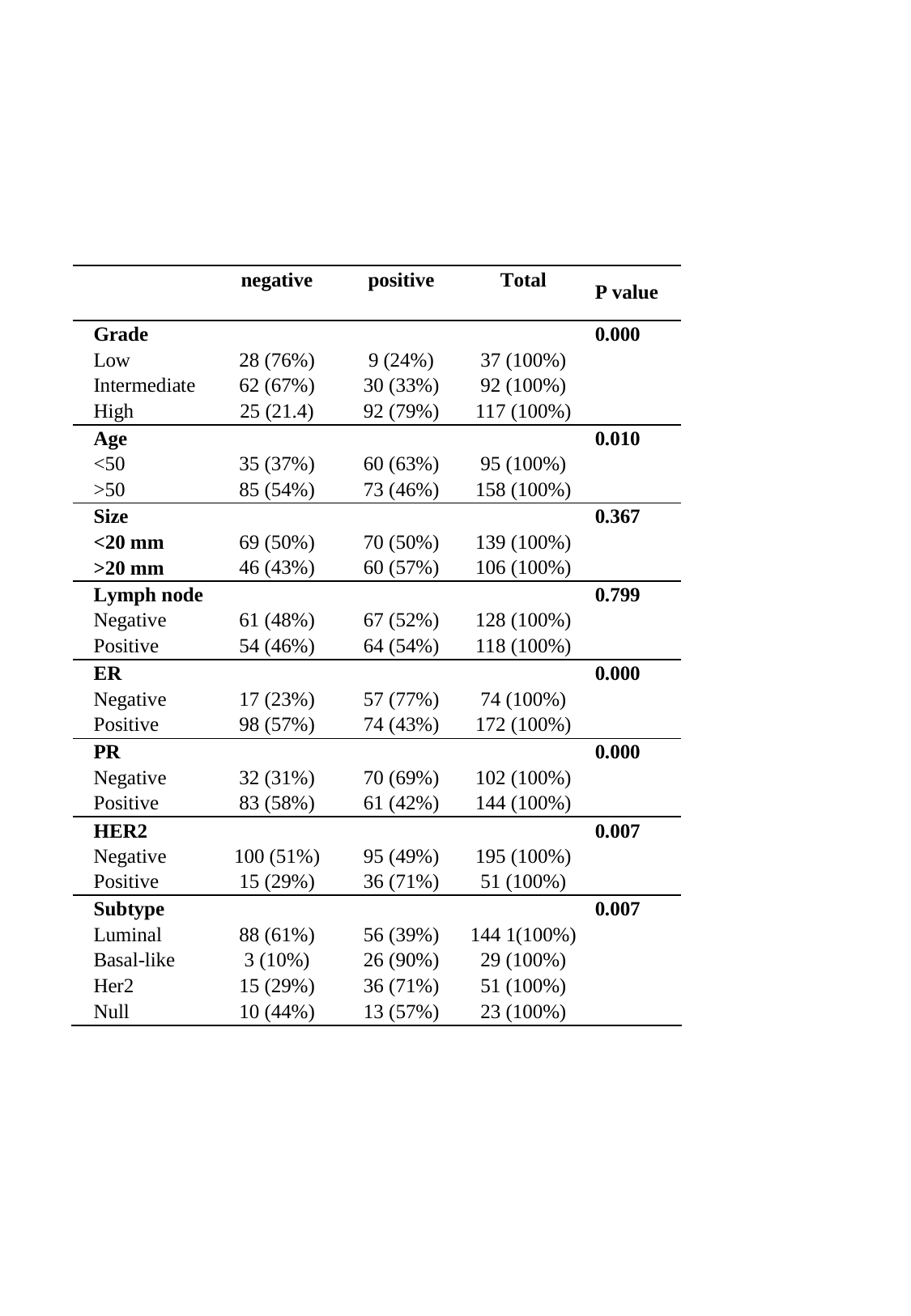

Supplement: Additional file 5 — Supplementary Table 5. Contingency table of seven in absentia homolog 2 (SIAH2) gene expression in invasive breast carcinomas of the initial cohort with clinicopathological parameters [file bcr2828-S5.PPT]

## Slide 1
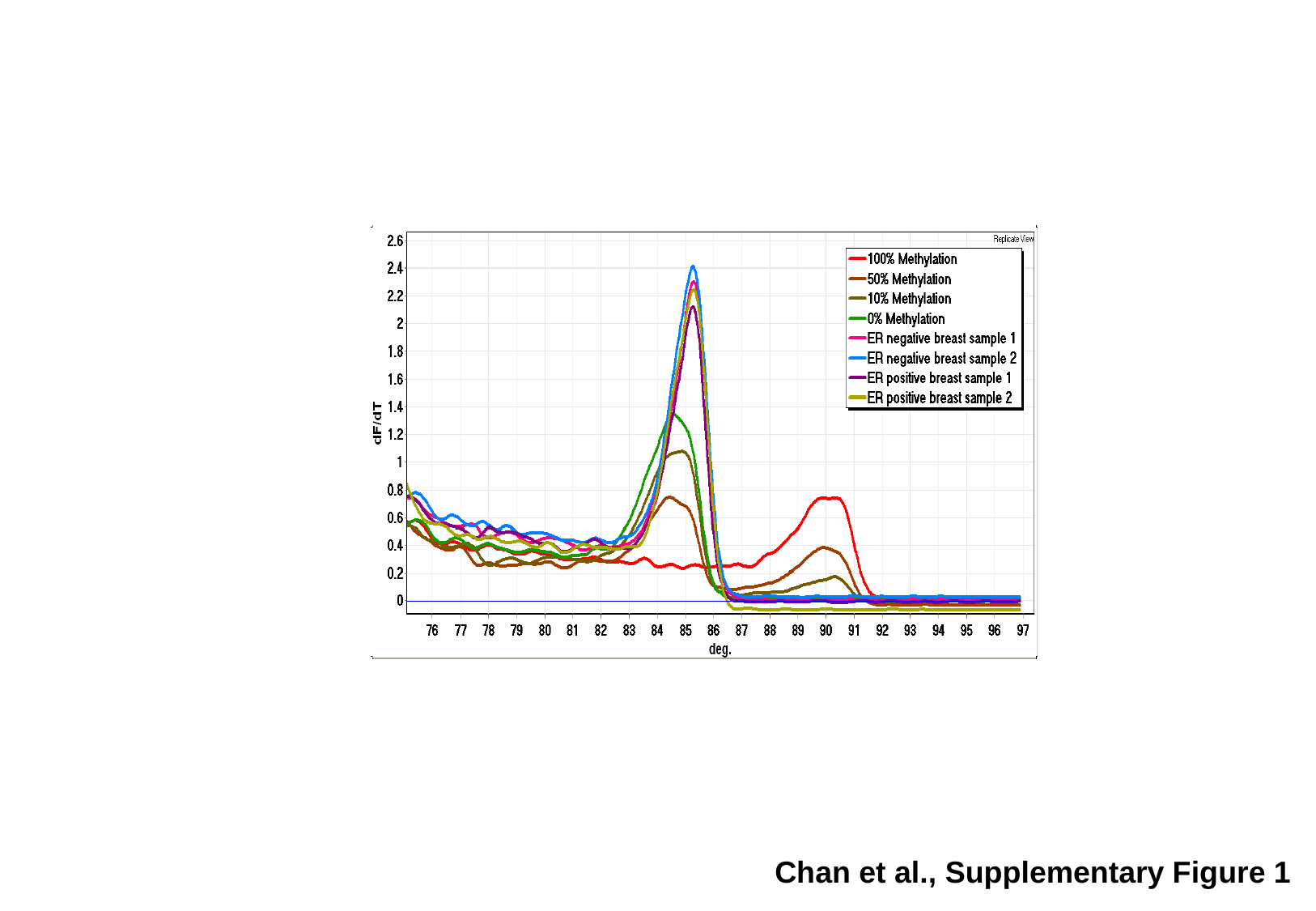

Chan et al., Supplementary Figure 1

Supplement: Additional file 6 — Supplementary Figure 1. Seven in absentia homolog 2 (SIAH2) gene methylation in breast carcinoma samples. Methylation-sensitive high-resolution melting (MS-HRM) detects sample methylation status by melting the amplicons after polymerase chain reaction assay. Methylated samples melt later than unmethylated samples, as they have cytosines in their sequences rather than the thymines after the bisulfite modification. Two estrogen receptor (ER)-positive and two ER-negative breast carcinomas show no methylation in SIAH2. Standard controls of 100%, 50%, 10% and 0% methylation are shown. The curve for each sample represents data from duplicate samples. [file bcr2828-S6.PPT]
